# The prediagnostic phase of Parkinson's disease

**DOI:** 10.1136/jnnp-2015-311890

**Published:** 2016-01-11

**Authors:** Alastair John Noyce, Andrew John Lees, Anette-Eleonore Schrag

**Affiliations:** 1 Department of Molecular Neuroscience, Reta Lila Weston Institute for Neurological Studies, UCL Institute of Neurology, London, UK; 2 Department of Clinical Neuroscience, UCL Institute of Neurology, London, UK

**Keywords:** PARKINSON'S DISEASE, EPIDEMIOLOGY, MOVEMENT DISORDERS

## Abstract

The field of prediagnostic Parkinson's disease (PD) is fast moving with an expanding range of clinical and laboratory biomarkers, and multiple strategies seeking to discover those in the earliest stages or those ‘at risk’. It is widely believed that the highest likelihood of securing neuroprotective benefit from drugs will be in these subjects, preceding current point of diagnosis of PD. In this review, we outline current knowledge of the prediagnostic phase of PD, including an up-to-date review of risk factors (genetic and environmental), their relative influence, and clinical features that occur prior to diagnosis. We discuss imaging markers across a range of modalities, and the emerging literature on fluid and peripheral tissue biomarkers. We then explore current initiatives to identify individuals at risk or in the earliest stages that might be candidates for future clinical trials, what we are learning from these initiatives, and how these studies will bring the field closer to realistically commencing primary or secondary preventive trials for PD. Further progress in this field hinges on greater clinical and biological description, and understanding of the prediagnostic, peridiagnostic and immediate postdiagnostic stages of PD. Identifying subjects 3–5 years before they are currently diagnosed may be an ideal group for neuroprotective trials. At the very least, these initiatives will help clarify the stage before and around diagnosis, enabling the field to push into unchartered territory at the earliest stages of disease.

## Introduction

**Figure 1:**
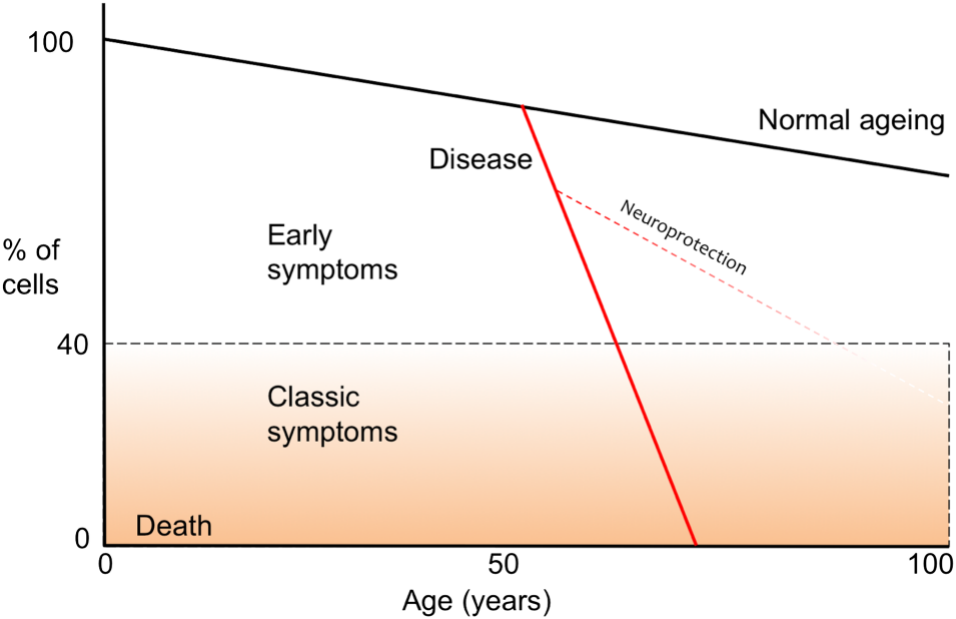
A schematic depicting normal (black solid line) and Parkinson's disease-related (grey solid line) nigral cell loss over time, including the point at diagnosis typically occurs (horizontal black hashed line) and the potential for modifying the trajectory of degeneration, if identified earlier (hashed grey line). NB. For colour version grey lines appear red, black lines remain black.

The motor features of Parkinson’s disease (PD) (tremor, rigidity, slowness and balance problems) are identified relatively late in the pathological process when approximately 50% of dopaminergic neurons have been lost in the substantia nigra. Symptomatic treatment is efficacious, but there are currently no drugs that demonstrably slow the disease course. It is believed, albeit not proven, that pathology may be too far advanced at the point of clinical diagnosis to be affected by potentially neuroprotective treatments (assuming that these are available). Identifying individuals at the earliest stages of disease would pave the way for clinical trials of emerging and repurposed drugs to prevent/delay progression to clinically manifest PD (see figure 1). However, modifying risk in those that do not yet have a diagnosis represents a challenge. The terms ‘early disease’ or ‘at-risk’ are frequently used synonymously due to uncertainty about the point at which the pathological process starts, but clarification will be important since it will determine whether prevention is attempted on a primary or secondary basis, and factors that initiate pathology may not necessarily be the same as those that subsequently drive progression. Inability to identify disease activity before diagnosis precludes distinction of the two, but this limitation may be overcome, given current momentum in the field of biomarkers.

In this review, we describe current knowledge and emerging findings in the prediagnostic phase of PD, including early features, genetic and environmental risk and protective factors, discuss current strategies to identify individuals at earliest disease stages to include in future clinical trials, and highlight how the knowledge gleaned from these studies might bridge the gap into preventive or protective drug trials for PD.

## Identification of individuals at the earliest stages

### Genetic factors

**Figure 2:**
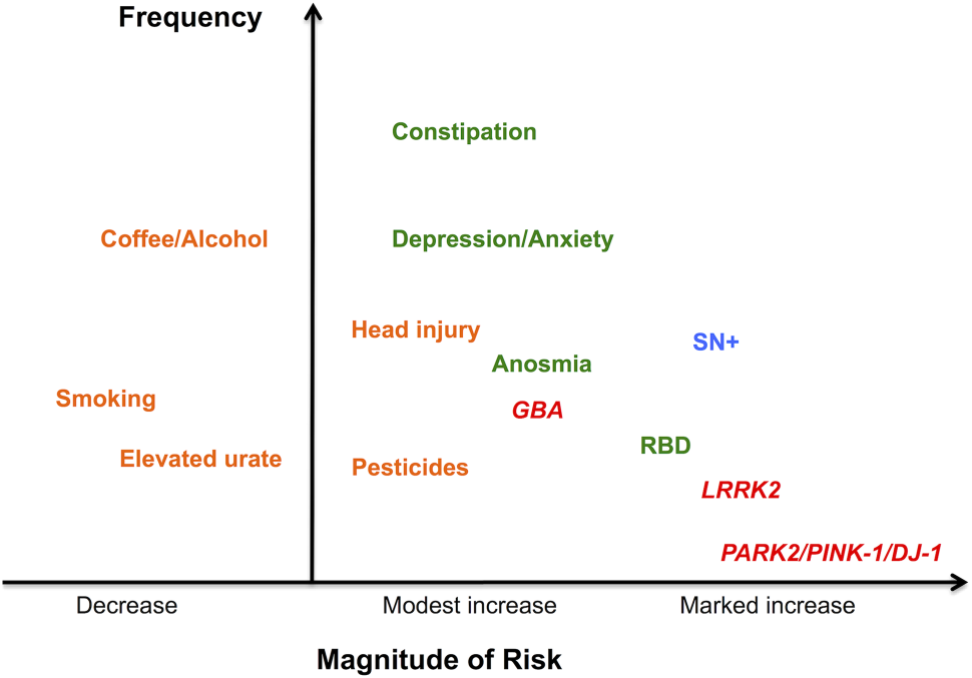
Risk factors and early features of Parkinson's disease associated with increased (or decreased) risk of subsequent diagnosis. Estimated magnitude of effect is plotted against estimated frequency. SN+ is hyperechogenicity in the region of the substantia nigra using transcranial sonography. Genetic and environmental risk factors are shown in lighter grey, and prediagnostic features and hyperechogenicity in darker grey. NB. For colour version genetic factors are red, environmental factors are orange, pre-diagnostic features are green and imaging features are blue.

Having a family history of PD increases the odds of PD by 3–4.5-fold, and up to 10% of patients report a family history of PD.^1^ Studies into the genetic basis of PD implicate lysosomal and mitochondrial dysfunction, and inflammation in pathogenesis.^2^ ^3^ Of the confirmed monogenic forms of PD, most result in abnormalities of one or more of these processes, but most are exceedingly rare and do not account for elevated risk at a population level (see figure 2). A central player in the disease is α-synuclein and mutations in the *SNCA* gene, which encodes this protein, are a cause of familial PD. Intraneuronal accumulation of α-synuclein is the pathological hallmark of PD, and mounting evidence suggests that fibrillar and oligomeric forms of the protein may be neurotoxic.^4^ ^5^ The full picture of how these complex processes combine to result in neurodegeneration remains incomplete, but current theories include the possibility of prion-like cell-to-cell propagation.^6^

Mutations in the *LRRK2* gene are the commonest known genetic cause for PD, and the G2019S mutation occurs in 4% of hereditary and 1% of sporadic PD.^7^ *LRRK2*-related disease has age-dependent penetrance (28% at 59 years, 51% at 69 years and 74% at 79 years), meaning that only a proportion of carriers will develop PD during life.^7^ *LRRK2* mutation carriers have been shown to have subclinical dopaminergic abnormalities, measured with functional imaging, and higher rates of non-motor features of PD than non-carriers.^8^ ^9^ In patients with PD and *LRRK2* mutations, the motor picture may be similar to idiopathic PD, but wider manifestations may not be.^7^ ^8^

Heterozygous mutations in the glucocerebrosidase (*GBA*) gene are associated with an increased risk of PD. Large studies have shown that *GBA* mutations are common in Ashkenazi Jews, occurring in 15% of patients and 3% of controls.^10^ In unselected patients with PD, 3.5% carry disease-associated *GBA* mutations compared with <1% of controls.^10^ ^11^ Clinical features of patients with PD and *GBA* mutations may be similar to sporadic PD and generally respond to levodopa, but onset of parkinsonism can be earlier, and carriers tend to have higher rates of cognitive problems and R(apid)E(ye)M(ovement) sleep behaviour disorder (RBD).^11^ Furthermore, early non-motor features of PD such as depression, subjective RBD and olfactory dysfunction have been observed in carriers of *GBA* mutations without PD, when compared with healthy controls.^12^

The study of manifesting and non-manifesting carriers of *LRRK2* and *GBA* mutations is important for understanding the prodromal phase of PD, and for studies of drugs targeting specific pathways. Cohorts of these subjects have been assembled to fulfil these aims, but with greater study, further differences may emerge between PD related to these mutations and sporadic disease. Other examples of monogenic PD include mutations, duplications and triplications of the *SNCA* gene causing dominantly inherited PD, and mutations in *PARK2*, *PINK1* and *DJ1* causing recessively inherited forms of PD (for review see ref. 3), with more emerging gradually. These monogenic causes of PD are too rare to base predictive studies on, but they give important insight into disease mechanisms and therapeutic targets.

Mutations in single genes do not account for all the heritable risk apparent in complex diseases, and genome-wide association studies, in which large numbers of unrelated cases are compared with unrelated controls, have yielded informative results. There are at least 28 independent risk variants associated with PD that increase or decrease risk in a small but potentially additive way.^13^ This enables the construction polygenic risk profiles, pooling the combined effect of multiple variants to estimate risk of PD or indeed age of disease onset.^14^ ^15^ However, the heritable component of PD is estimated to be greater still, at around 30%, and identified risk loci and monogenic forms explain only about 5–10%.^16^ Over time, with increasing numbers of studied cases and controls, along with deep resequencing and precision phenotyping, a greater proportion of the heritability of PD will be uncovered. The influence that genetic variation has on PD is not limited to risk of getting disease alone, and specific variants are likely to contribute additionally to age of disease onset, progression and phenotype, with a number of indicative studies reported thus far.^11^ ^17^ Furthermore, these and additional genetic factors may dictate therapeutic choices in the future in the clinical setting and in terms of recruitment to clinical trials. The genetic architecture of PD is continually expanding and increasingly complex. It has implications for multiple aspects of the disease, but other factors are also important in determining risk.

### Environmental risk factors

There is a large body of evidence demonstrating a small but significantly elevated risk of PD associated with a number of environmental risk factors (see figure 2). Some of the strongest environmental evidence exists for exposure to pesticide and proxies for this, including farming occupation, rural living and drinking well water.^1^ Specific implicated pesticides include rotenone and paraquat (structurally related to 1-methyl-4-phenyl-1,2,3,6-tetrahydropyridine (MPTP), which has also been linked to parkinsonism in users of illicit drugs^18^), and both these chemicals are used to create animal models of PD. Other potential toxins include heavy metals such as manganese, with exposure arising through occupations such as welding and in recreational ephedrone users.^19^ It seems unlikely that environmental toxins play more than a minor role in PD risk overall, with meta-analysis suggesting ORs in the region of 1.2–2.0.^1^

Pooled observational study data also implicates head injury as a minor but significant risk factor for PD.^1^ ^20^ There is increasing evidence that individuals who suffer recurrent head injury, particularly sportspersons such as boxers, jockeys, American football and rugby players, are at risk of developing a range of degenerative neurological conditions including parkinsonism, dementia and motor neuron disease, although pathological examination of these subjects tends to reveal alternative pathology than that typically associated with PD.^21^

By stark contrast with other common chronic diseases, there exist a number of intriguing but consistent negative associations with PD and lifestyle factors such as smoking, caffeine and alcohol.^1^ Additionally, there are a number of medications for which negative associations with PD have been reported in observational studies, including calcium channel blockers, non-steroidal anti-inflammatories and statins.^1^ Some of these are currently the focus of clinical trials examining their potential use in PD.

Whether these exposures offer true neuroprotective properties or whether negative association, at least with lifestyle factors, arises due to a common feature (eg, avoidance as part of an early PD personality change) is yet to be determined. The former possibility is supported by clinical studies reporting improvement of motor function in a clinical trial of caffeine to treat excessive daytime somnolence in PD and PD animal models that show protective effects of nicotine on nigrostriatal damage.^22^ ^23^ Spurious association may arise as a result of reverse causality, a potential problem with observational studies. This may be plausible even in prospective studies that exclude cases of incident PD in the first few years of follow-up because the prodromes of PD are likely to be very long, during which time pathology is present, but the classical features are not yet overt.

Another consistent negative association exists between levels of serum urate and PD, with a number of studies demonstrating a ‘protective’ effect of elevated serum urate.^1^ The most recent of these studies is an example of Mendelian randomisation (MR), which is a powerful technique to assess casual relationships between risk factors and disease. In MR studies, a gene variant is used as a proxy for an environmental exposure (or intermediate phenotype), to determine the effect of this on a disease outcome. Simon and colleagues used single nucleotide polymorphisms in the *SLC2A9* gene (which explain a proportion of the genetically specified variability in serum urate) to test whether these polymorphisms predicted rate of progression in early PD. They demonstrated that *SLC2A9* status was associated with elevated serum urate and was associated with slower disease progression.^24^ The MR approach is protected against biases that can arise in traditional observational studies assessing causation; if, for example, reduction in serum urate occurred as an early consequence of PD, a spurious negative association between the exposure and outcome might arise. MR on the other hand operates more like a randomised controlled trial in which the exposure (in this case a gene variant) is randomly allocated at baseline (conception) along with known and unknown confounding factors. Given the consistency of this relationship in the MR study with previous observational studies, therapeutic modulation of urate levels is a strong target for clinical trials.

Increasing research activity in exploring the overlap between genes and the environment will further our understanding of the causal basis of disease. As for many diseases, the total picture of risk remains incomplete due to apparent and substantial randomness of the onset, the obscuration of risk factors either because of rarity, ubiquity or poor measurement, or the fact that disease tends to strike those at moderate risk, simply because those at ‘highest risk’ are far fewer.

### Early clinical features

Recognition of the importance of non-motor features of PD has been building for several years.^25^ ^26^ In established PD, patients regard non-motor symptoms at least as troublesome as motor features, and treatment is often difficult.^27^ Non-motor symptoms are experienced early and there is substantial evidence which suggests that they also predate diagnosis by several years (see figure 2). A number of studies have demonstrated the association of PD with earlier diagnoses such as depression, anxiety, constipation and erectile dysfunction.^1^ ^28^ ^29^

The best characterised early non-motor features are idiopathic anosmia and RBD. Anosmia is relatively common in the ageing population and is non-specific, with only a proportion going on to develop neurodegenerative disease.^30^ RBD on the other hand, is relatively specific for neurodegeneration, with high conversion rates, but clinical and pathological heterogeneity in that it can precede PD, dementia with Lewy bodies and multiple system atrophy.^31^ An important distinction lies between subjective RBD (on clinical history or questionnaire-based diagnosis) and that which is formally diagnosed with an overnight sleep study and polysomnography (PSG). PSG-confirmed RBD is rare in the general population, and the largest observational study amassed just over 300 subjects despite international collaborative efforts.^32^ Nonetheless study of PSG-confirmed RBD has resulted in positive strides forward given that time during which a proportion will convert to neurodegenerative disease is known (25% at 3 years and 40% at 5 years).^32^ The emergence of anosmia or subtle motor signs, in particular, in those with RBD appears to further refine estimates of those that are likely to convert.^33^ ^34^

Characterisation of non-motor features is potentially valuable for early identification of PD and the Movement Disorders Society (MDS) has recently released Research Criteria for Prodromal PD.^35^ The literature initially described the early phase of PD as being the ‘pre-motor’ phase, but more recently this has fallen out of favour with the recognition that subtle motor features can be present before diagnosis.^28^ ^36^ The clinical diagnosis of PD requires multiple motor features to be established, and while subtle motor signs may be present, a clinical diagnosis of PD cannot be made until they become more definite.^4^ Given that subtle or single motor abnormalities occur prior to diagnosis and alongside early non-motor features, this period is better referred to as the prediagnostic phase.^28^ The MDS recommends the following terminology:^35^

1. Preclinical PD—presence of neurodegenerative synucleinopathy without clinical symptoms (this stage will be defined by disease biomarkers when available).
2. Prodromal PD—presence of early symptoms and signs before PD diagnosis is possible.
3. Clinical PD—diagnosis of PD has been made based on the presence of classical motor signs.

The emergence of large longitudinal primary care datasets has allowed detailed exploration of the full range of early motor and non-motor symptoms that predate PD, while being free from the biases that many traditional observational studies have suffered from.^28^ Alongside, advances in wearable technology, and the availability of remote testing, will aid objective measurement of emerging motor dysfunction in those at risk of PD.^37^ A variety of measuring devices have been developed, including software applications which harness information on activity and motion (and in some cases speech) captured by smart phones and tablet-devices, custom-built sensors that measure gait, bradykinesia, dyskinesia and nocturnal movements, keyboard-based speed and accuracy tests, as well as custom-built home-testing devices (for examples of these, see online supplementary material). Many of these devices demonstrate high sensitivity and specificity in differentiating patients with PD from controls, and are likely to see increasing use in clinical practice to guide decision-making. Despite the indication that objective motor dysfunction occurs prior to diagnosis in PD, currently there are few examples of the application of remote or wearable devices in prediagnostic PD. A major foreseeable hurdle is ensuring that validation and regulatory approval for software and devices will keep abreast of rapid and continuous change in available technology.^38^

### Imaging markers

Radiotracer imaging with single photon emission computed tomography (SPECT) or positron emission tomography (PET), and transcranial sonography (TCS) have repeatedly demonstrated the ability to differentiate patients with PD from healthy individuals with adequate sensitivity and specificity (see table 1).^39^ These modalities may also have the potential to identify subclinical PD prior to diagnosis, and early support for this notion came from studies showing that SPECT and PET were typically abnormal bilaterally in patients with unilateral PD, and could identify unaffected twins of patients with PD who later went on to develop PD.^39^ Using SPECT, the clinical diagnosis of PD tends to be made once there has been 40–50% reduction in tracer binding and an average 11.2% decline in striatal binding has been observed each year after diagnosis.^39^ Multitracer PET has also shown deterioration over time in patients with established PD, but is expensive when considering use on a large scale.^40^

**Table 1:** Imaging modalities and markers for Parkinson's disease

Modality	Example tracers/sequence	Observation(s)	Analysis	Accessibility	Cost	Suitability for screening
SPECT	^123^I-β-CIT ^123^I-FP-CIT	Loss of binding in striatum	Visual inspection, quantification	++	+++	+++
TCS	2–3.5 Hz transducer	Hyperechogenicity in the region of the substantia nigra	Visual inspection, quantification	++++	+	+++
PET	^18^F-dopa ^18^F-FDG	Loss of binding in striatum May help differentiate atypical PD	Visual inspection, quantification	++	++++	+
MRI	Traditional (T1& T2), T2/T2* (gradient echo), DTI, spin echo, fMRI	Numerous reported, none established	Visual inspection, quantification	+++	++	++
MIBG	^123^I-meta-iodobenzylguanidine	Low heart-to-mediastinum ratio	Visual inspection, quantification	++	++	++

Accessibility, cost and suitability for screening are estimated semiquantitatively on a four-point relative scale with + lowest and ++++ highest.

DTI, diffusion tensor imaging; FDG, fludeoxyglucose; fMRI, functional MRI; Hz, hertz; MIBG, metaiodobenzylguanidine; PET, positron emission tomography; SPECT, single photon emission computed tomography; TCS, transcranial sonography.

Evidence of demonstrable longitudinal change using DaT (dopamine transporter) SPECT in early PD is now being sought with serial studies performed as part of the Parkinson's Progression Markers Initiative (PPMI) study.^41^ Furthermore, progressive change in SPECT imaging has been reported in a study of patients with RBD, and in the Parkinson's At Risk Study (PARS), which includes subjects with idiopathic anosmia (and other prodromal markers).^42^ ^43^

By contrast, TCS demonstrating hyperechogenicity of the substantia nigra (SN) appears to be a static rather than changing marker.^39^ A study in individuals with mild parkinsonian features, found a sensitivity of 91%, specificity of 82% and positive predictive value (PPV) of 93% for SN hyperechogenicity and PD diagnosis after follow-up, despite blinding those performing sonography to clinical details at baseline.^44^ Current efforts to improve standardisation and quantitative analysis for TCS and SPECT may increase their utility in the prediagnostic phase of disease, with SPECT more likely to demonstrate sensitivity to change.

High-field and novel sequences of MRI may also provide opportunities to address the challenge of imaging disease progression in the prediagnostic phase of PD. Correlations of MRI microstructural imaging abnormalities have been reported with postmortem findings and quantitative differences between patients with PD and healthy subjects in terms of iron deposition, loss of neuromelanin and alterations in nigrosome 1, in mainly single studies (for examples see online supplementary material). In addition to subtle structural abnormalities, application of functional MRI methods have shown differences in functional connectivity with the basal ganglia network, the default-mode network and the sensorimotor resting state network, between patients with PD and healthy controls, some of which are influenced by dopaminergic medication (see online supplementary material for examples of studies).

^123^I-meta-iodobenzylguanidine (MIBG) myocardial scintigraphy has been largely studied in Japan, with multiple reports showing a reduction in heart to mediastinum ratio of MIBG uptake in patients with PD, compared with healthy controls or other degenerative causes of parkinsonism.^45^ Cardiac sympathetic nerve involvement is a feature of incidental Lewy body pathology, which is believed to be the pathological precursor to PD.^46^ Altered MIBG uptake has been reported in patients with a range of early non-motor features of PD, including autonomic dysfunction, mood disorders and sleep disorders, meaning that it may be a good prediagnostic imaging marker for PD, but further studies are required.^47^

### Fluid and tissue biomarkers

Given the supposed central importance of α-synuclein to the disease process, recent biomarker strategies have centred on finding and characterising forms of the protein in a range of biofluids and tissues. Blood has been a disappointing target to-date because red cells contain large quantities of α-synuclein, obscuring any theoretical difference in levels between patients and controls, but some group differences have been demonstrated for DJ-1, urate, vitamin D and IGF-1 (for examples of individual studies see online supplementary material).^48^ Plasma apoliprotein A1 has been shown to differentiate patients from healthy controls and, interestingly, also to correlate with DaT SPECT deficit in hyposmic subjects with a family history of PD, recruited from the PARS study.^49^

It is believed that cerebrospinal fluid (CSF) is most likely to show changes representative of disease occurring in the brain. Nonetheless, lumbar puncture is an invasive and costly procedure compared with blood draw, and is likely to remain most appropriate for use in clinical trials and in specific clinical practice settings, unless a high-performing biomarker can be identified. Potential CSF biomarkers for PD include α-synuclein and DJ-1, with Aβ_42_ potentially correlating with cognitive impairment, and various forms of tau protein and neurofilament light-chain differentiating PD from atypical parkinsonian disorders.^50^ Separately, observed biomarker changes in saliva have included reduction in α-synuclein levels and elevation of DJ-1 in the saliva (see online supplementary material for examples of individual studies of CSF and salivary biomarkers).

α-Synuclein pathology is found outside the central nervous system in patients with PD (even preceding diagnosis in some instances), using a variety of sampling methods, immunohistochemical techniques, in fresh and archival tissue. The gut has been proposed as the site of initiation of PD pathology, but the burden of synucleinopathy correlates poorly with disease severity and the proximal (more) to distal (less) gradient in pathology appears at odds with this suggestion.^51^ ^52^ In addition, there is variability in the reporting of cell loss and a current lack of consensus of what should be classified as Lewy pathology given that the appearances in gut are dissimilar to what is seen typically in the brain of patients, and the fact that control subjects have variable staining for α-synuclein.^53^ The potential for gut biomarkers remains high, however, and may not be restricted to tissue sampling. Recently, interest has been stirred by demonstrable differences in the faecal microbiome of patients and controls.^54^

Salivary glands may also be a peripheral source for demonstrating PD-related pathology but, like gut biopsies, the invasive nature and cost of deep tissue sampling or full colonoscopy currently make both undesirable.^52^ ^55^ Of significant interest is a recent study showing synuclein deposition in dermal punch biopsies taken from patients with PD, and differentiating these subjects from other forms of parkinsonism.^56^ While these findings require replication, the skin offers the exciting possibility of yielding biomarkers for PD, and is significantly more accessible than salivary glands and the gut. A summary of tissue and fluid biomarkers is provided in table 2.

**Table 2:** Tissue and fluid biomarkers for PD

Source	Medium	Main techniques	Candidate markers	Invasiveness	Cost	Suitability for screening
Cerebrospinal fluid	Fluid	Assay, electrophoresis, mass-spec	t-α-syn, p-α-syn, t-tau, p-tau, NFL	+++	++	++
Blood	Fluid	Assay, electrophoresis, mass-spec	Uric acid, inflammatory markers, IGF-1, ApoA1, DJ-1	++	+	+++
Saliva	Fluid	Assay, electrophoresis, mass-spec	α-syn, DJ-1	+	+	+++
Gut	Tissue biopsy	Immunohistochemistry	t-α-syn, p-α-syn	++++	+++	++
Salivary gland	Tissue biopsy	Immunohistochemistry	t-α-syn, p-α-syn	++++	+++	+
Skin	Tissue biopsy	Immunohistochemistry	t-α-syn, p-α-syn	+++	++	+++

Invasiveness, cost and suitability for screening are estimated semiquantitatively on a four-point relative scale with + lowest and ++++ highest.

ApoA1, apolipoprotein A1; IGF-1, insulin like growth factor 1; NFL, neurofilament light chain; p-α-syn, phosphorylated α synuclein; t-α-syn, total α synuclein.

## Current studies mapping the prediagnostic and peridiagnostic phases of PD

Several studies (table 3) have been initiated: to identify those in the prediagnostic and prodromal phases of PD; identify clinical and biological markers to track progression of disease before diagnosis; create platforms to find subjects for inclusion in neuroprotective drug trials. As outlined above, some initiatives recruit individuals with a single strong risk factor such as carrier status for *LRRK2* or *GBA* mutations, or idiopathic RBD or anosmia, in order that subjects may be followed prospectively, whereas other approaches employ large population-based cohorts or retrospective case–control methods to examine associations with PD and preceding diagnoses. From the former we learn more about the emergence of PD in specific risk cohorts, which, in turn, may prove to be appropriate for recruitment to clinical trials. They are likely to be more homogenous in terms of their disease mechanisms, pathology and clinical features, as well as being the simplest in which to determine ‘time to conversion’. However, they are perhaps not representative of the spectrum of PD as a whole, and biomarkers developed in these groups must be replicated in sporadic PD cohorts before assuming that they are applicable to all. The latter are difficult, and costly to conduct with in-depth assessments and appropriate sample sizes, but allow the investigation of risk/protective factors and early symptoms and signs that precede emergence of established PD. This, in turn, informs strategic combination of factors to try and delineate individuals at high risk while also capturing the full spectrum of PD. Although the magnitude of risk associated with individual risk factors and early non-motor features has been reported, the best combination of risk factors for predicting PD remains unknown.^1^ ^35^ Several studies are now seeking to combine risk factors for PD in order to improve predictive power with which those at increased risk of PD can be identified, with and without imaging markers.

**Table 3:** Studies in the prediagnostic phase of Parkinson's disease

Acronym	Study name	Participants	Country	Number recruited	Tests/exposures	Outcome
PRIPS^57^	Prospective validation of risk factors for the development of Parkinson syndromes	Subjects over 50 years old	Germany, Austria	1847	TCS, smell, UPDRS	Clinical diagnosis of PD
TREND^58^	Tübinger evaluation of risk factors for the early detection of neurodegeneration	Subjects over 50 years with anosmia, self-report RBD or depression	Germany	>1200	TCS, smell, UPDRS, quantitative motor, psychometry, blood biomarkers	Clinical diagnosis of PD
PARS^43^	Parkinson's at-risk syndrom study	Subjects over 50 years with hyposmia and DaT deficit on SPECT	USA	4999 (completed baseline smell test)	Smell, DaT SPECT, UPDRS, cognition, blood biomarkers	Clinical diagnosis of PD/DaT deficit on SPECT
P-PPMI^41^	Prodromal Parkinson's Progression Markers Initiative	Subjects with prodromal features or gene mutations	International	Anosmia/RBD=65 Genetic=150	CSF and blood biomarkers, UPDRS, cognition, sleep and autonomic assessments	Clinical diagnosis of PD
OPDC^59^	Oxford Parkinson's Disease Centre study	Subjects with a first-degree relative with PD, or subjects with RBD (PSG confirmed)	UK	∼190	UPDRS, non-motor assessments, blood and CSF biomarkers	Clinical diagnosis of PD
PREDICT-PD^60^	PREDICT-PD	Subjects over 60 years old	UK	1323	In all (online): risk scoring, smell, RBDSQ, quantitative motor (BRAIN test), genetics In extremes of risk: UPDRS, cognitive, TCS	Clinical diagnosis of PD/DaT deficit on SPECT

BRAIN test, BRadykinesia Akinesia INcoordination test; B-SIT, Brief Smell Identification Test; CSF, Cerebrospinal Fluid; DaT, Dopamine Transporter; PD, Parkinson's disease; PSG, polysomnography; RBD, REM sleep Behaviour Disorder; RBDSQ, RBD Screening Questionnaire; SPECT, single photon emission computed tomography; TCS, Transcranial Sonography; UPDRS, Unified Parkinson's Disease Rating Scale.

The Prospective validation of Risk factors for the development of Parkinson Syndromes (PRIPS) study was a large prospective study that sought to determine the magnitude of risk of PD that SN hyperechogenicity conveyed.^57^ Over 1800 participants were screened and 304 had hyperechogencity. At 3 years follow-up, 11 had developed PD and the relative risk of incident PD was 17.3 (95% CI 3.7 to 81.3) if there was SN hyperechogenicity at baseline.^57^ The aforementioned PARS study uses objective smell testing to identify subjects with idiopathic anosmia at stage 1, followed by DaT SPECT at stage 2 to identify subclinical presynaptic denervation.^43^ The study has reported early results which demonstrated reductions in nigrostriatal DaT binding in subjects with hyposmia compared to those with normal smell, as well as associations between a number of prodromal features of PD within the study cohort.^43^ The Tübinger evaluation of Risk factors for the Early detection of NeuroDegeneration (TREND) study examines subjects who are over 50 years of age, with a limited combination of risk factors (anosmia or depression or RBD), using serial studies of movement, laboratory tests and imaging, with follow-up to incident PD. Baseline data from this cohort have been reported showing associations between selected prodromal markers and other early associated features of PD.^58^

Two large multicentre studies, one coordinated from the USA (the Parkinson’s Progression Markers Initiative, PPMI) and one based in the UK (the Tracking Parkinson’s or PRoBaND study), recruit patients immediately after the clinical diagnosis of PD and undertake detailed clinical, imaging and biomarker studies longitudinally. While not strictly looking at prediagnostic PD, the PPMI and PRoBaND studies will help define the role of clinical markers (motor and non-motor) in the early measurement of PD, and the identification of novel imaging and laboratory biomarkers, as well as giving insight into what might be apparent through back-extrapolation to the prediagnostic phase. The PPMI study also includes a prodromal arm (P-PPMI) in which subjects with RBD, anosmia or a mutation *(LRRK2, GBA or SNCA*), will be assessed and followed in the same way as PD subjects, allowing for a seamless examination of the prediagnostic and early disease stages of PD. Separately, as part of a large study aimed at understanding the biological basis of disease in patients with established PD, the Oxford Parkinson's Disease Centre (OPDC) includes smaller ‘high-risk groups’ with a family history or RBD. Clinical assessments, laboratory and imaging biomarker studies are being undertaken and early results are emerging.^59^

In the UK, the PREDICT-PD study combines risk factors and early non-motor features to devise a risk scoring process for future PD. Risk scores were calculated based on a meta-analysis of the published literature.^1^ These, in turn, were used to generate ORs for the effect on risk of PD ascribed by individual early non-motor features and risk factors. Using *a priori* odds of PD attributed to age, a Bayesian model of risk was constructed to yield combined risk estimates for each subject in the study.^60^ The study runs almost entirely via the internet with more detailed laboratory, motor and imaging investigation for groups at the extremes of risk. PREDICT-PD is the first study to try and combine large numbers of risk factors for PD and has the potential to screen a large, community-based population, and aims to facilitate recruitment into clinical trials in the future. Unlike some of the other studies, it seeks to identify individuals spanning the full spectrum of PD, which makes this cohort highly applicable to occurrence of typical PD in clinical settings.

## Further challenges and opportunities in the prediagnostic phase

**Figure 3:**
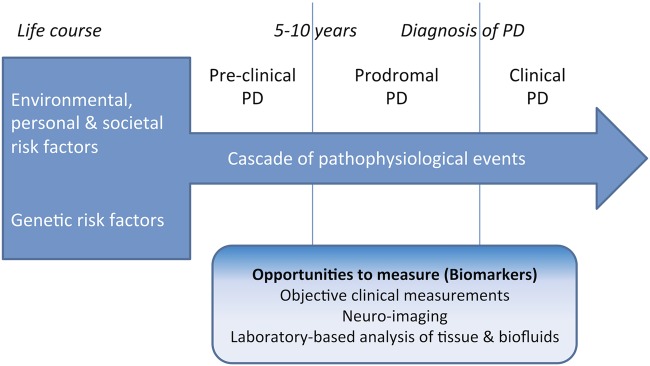
A schematic showing determinants of risk, the prediagnostic phase (preclinical and prodromal phases) and clinical phase of Parkinson's disease, along with the parallel application of risk and disease progression markers to measure disease activity across phases.

The above studies aim to overcome the important challenge of identification of ‘at-risk’ individuals who may develop the classical clinical syndrome of PD, with the eventual aim of initiating treatment to avoid or delay clinically relevant symptoms. In addition, studies such as PPMI (with P-PPMI), TREND, PARS and PREDICT-PD will document the time immediately before, during and after the emergence of clinically recognisable PD, delineating the clinical and biomarker features of this phase that will be crucial to commencing clinical trials. These studies will help refine the determination of risk status and course of early disease progression (see figure 3).

There are additional hurdles that must be overcome before clinical trials recruiting subjects in this phase of disease can be planned: (1) determination of appropriate study endpoints and duration of trials. Prevention or delaying emergence of classical symptom onset is the ultimate aim, but PD is insidious, with its clinical manifestations emerging over months and years, making many clinical end points unsuitable and such studies difficult to fund for the duration required. A sensitive clinical marker of progression would be valuable in detecting subtle changes at this early phase, however, an imaging or laboratory marker that spanned the prediagnostic and early postdiagnostic phases may offer better sensitivity, specificity, reliability and precision overall (see figure 3). This, in turn, could allow appropriate calculation of sample sizes and trial duration, dependent on anticipated drug effect; (2) another important consideration in trial design is the heterogeneity in clinical manifestation of PD, rate of progression and the presence/absence of other features. Clinical trials designed to show the disease-modifying effect of an agent may initially need to include homogenous samples or samples stratified for presentation and rate of progression in order to show an effect before trials in wider groups can be conducted; (3) other factors are continuity and applicability through the early stages of the disease. Even with an optimised early detection process, there will still be individuals who are ‘undetected’, and first present with overt signs of PD, and potential treatments will need to be assessed for demonstrable effects in these subjects too. Longer-term observational studies that examine risk status could support registries through which subjects indicate their willingness to participate in future clinical trials and biomarker initiatives. Consenting eligible subjects could be offered inclusion into clinical trials with the benefit of extensive available pre-trial data, but issues of selection bias and generalisability of results must be considered; (4) Finally, there are ethical implications of treating at-risk populations. For a repurposed drug, with previous data on safety and tolerance, the implications of undertaking clinical trials in those at-risk are perhaps less than for novel drugs with unknown safety profiles and potential toxicity. Justification for more invasive therapies could probably not be found without clear results in established PD. In addition, disclosure of risk status is likely to be a prerequisite for participation in clinical trials, but has the potential to bias recruitment and poses an ethical barrier in the absence of proven neuroprotective effects. Ultimately, disclosure may be unavoidable in order to make an informed decision about trial participation.

## Conclusion

Significant progress has been made in the understanding and identification of subjects in the prediagnostic phase of PD and a number of initiatives are underway to further define these groups. These studies may contain subjects that would be candidates for recruitment into clinical trials targeting neuroprotection within a few years. Parallel exploration of peripheral tissue, fluid and multimodal imaging is needed to identify differences between patients and controls across a range of markers. Of major interest is whether these differences can be demonstrated in high-risk/prediagnostic subjects, and whether they change up to the point of diagnosis and immediately beyond. This will enable testing of drug therapies at a time when more neuronal tissue can potentially be preserved, and there is an absence of symptomatic effects of medication with the potential to confound.

## Supplementary Material

Web supplement
