# Supplementary material for: The prediagnostic phase of Parkinson's disease
Source: J Neurol Neurosurg Psychiatry. 2016 Jan 11;87(8):871–8. doi: 10.1136/jnnp-2015-311890 (PMC4975823; doi:10.1136/jnnp-2015-311890)
Supplement: Web supplement [file jnnp-2015-311890-s1.pdf]

Supplementary file 1 – Additional individual published studies relevant to pre-diagnostic Parkinson's disease

Quantitative motor testing

1. Arora S, Venkataraman V, Zhan A, et al. Detecting and monitoring the symptoms of Parkinson's disease using smartphones: A pilot study. Parkinsonism Relat Disord 2015;21:650–3.
2. Pan D, Dhall R, Lieberman A, et al. A Mobile Cloud-Based Parkinson's Disease Assessment System for Home-Based Monitoring. JMIR mHealth uHealth 2015;3:e29.
3. Griffiths RI, Kotschet K, Arfon S, et al. Automated assessment of bradykinesia and dyskinesia in Parkinson's disease. J Parkinson Dis 2012;2:47–55.
4. Louter M, Maetzler W, Prinzen J, et al. Accelerometer-based quantitative analysis of axial nocturnal movements differentiates patients with Parkinson's disease, but not high-risk individuals, from controls. J Neurol Neurosurg Psychiatry 2015;86:32–7.
5. Tzallas A, Tsipouras M, Rigas G, et al. PERFORM: A System for Monitoring, Assessment and Management of Patients with Parkinson's Disease. Sensors 2014;14:21329–57.
6. Noyce AJ, Nagy A, Acharya S, et al. Bradykinesia-Akinesia Incoordination Test: Validating an Online Keyboard Test of Upper Limb Function. PLoS ONE 2014;9:e96260.
7. Goetz CG, Stebbins GT, Wolff D, et al. Testing objective measures of motor impairment in early Parkinson's disease: feasibility study of an at-home testing device. Mov Disord 2009;24:551–6.

Magnetic resonance imaging markers in Parkinson's disease

1. Reiter E, Mueller C, Pinter B, et al. Dorsolateral nigral hyperintensity on 3.0T susceptibility-weighted imaging in neurodegenerative Parkinsonism. Mov Disord 2015;30:1068–76.
2. Schwarz ST, Afzal M, Morgan PS, et al. The "swallow tail" appearance of the healthy nigrosome – a new accurate test of Parkinson's sisease: a case-control and retrospective cross-sectional MRI study at 3T. PLoS ONE 2014;9:e93814.
3. Blazejewska AI, Schwarz ST, Pitiot A, et al. Visualization of nigrosome 1 and its loss in PD: pathoanatomical correlation and in vivo 7 T MRI. Neurology 2013;81:534–40.
4. Peran P, Cherubini A, Assogna F, et al. Magnetic resonance imaging markers of Parkinson's disease nigrostriatal signature. Brain 2010;133:3423–33.
5. Ohtsuka C, Sasaki M, Konno K, et al. Differentiation of early-stage parkinsonisms using neuromelanin-sensitive magnetic resonance imaging. Parkinsonism Relat Disord 2014;20:755–60.
6. Szewczyk-Krolikowski K, Menke RAL, Rolinski M, et al. Functional connectivity in the basal ganglia network differentiates PD patients from controls. Neurology 2014;83:208–14.
7. Tessitore A, Esposito F, Vitale C, et al. Default-mode network connectivity in cognitively unimpaired patients with Parkinson disease. Neurology 2012;79:2226–32.
8. Esposito F, Tessitore A, Giordano A, et al. Rhythm-specific modulation of the sensorimotor network in drug-naive patients with Parkinson's disease by levodopa. Brain 2013;136:710–25.

Wet biomarker studies in Parkinson's disease

1. Godau J, Herfurth M, Kattner B, et al. Increased serum insulin-like growth factor 1 in early idiopathic Parkinson's disease. J Neurol Neurosurg Psychiatry 2010;81:536–8.
2. Lin X, Cook TJ, Zabetian CP, et al. DJ-1 isoforms in whole blood as potential biomarkers of Parkinson disease. Sci Rep 2012;2:954.
3. Ding H, Dhima K, Lockhart KC, et al. Unrecognized vitamin D3 deficiency is common in Parkinson disease: Harvard Biomarker Study. Neurology 2013;81:1531–7.
4. Schwarzschild MA, Schwid SR, Marek K, Watts A, Lang AE, Oakes D, et al. Serum urate as a predictor of clinical and radiographic progression in Parkinson disease. Arch Neurol 2008;65(6):716–23.
5. Chen-Plotkin AS, Hu WT, Siderowf A, et al. Plasma epidermal growth factor levels predict cognitive decline in Parkinson disease. Ann Neurol 2010;69:655–63.
6. Zetterberg H, Petzold M, Magdalinou N. Cerebrospinal fluid α-synuclein levels in Parkinson's disease - changed or unchanged? Eur J Neurol 2014;21(3):365–7.
7. Hong Z, Shi M, Chung KA, et al. DJ-1 and α-synuclein in human cerebrospinal fluid as biomarkers of Parkinson's disease. Brain 2010;133:713–26.
8. Parnetti L, Castrioto A, Chiasserini D, et al. Cerebrospinal fluid biomarkers in Parkinson disease. Nat Rev Neurol 2013;9:131–40.
9. Devic I, Hwang H, Edgar JS, et al. Salivary α-synuclein and DJ-1: potential biomarkers for Parkinson's disease. Brain 2011;134:e178–8.
10. Masters JM, Noyce AJ, Warner TT, et al. Elevated salivary protein in Parkinson's disease and salivary DJ-1 as a potential marker of disease severity. Parkinsonism Relat Disord 2015;21:1251–5.
11. Kang WY, Yang Q, Jiang WF, et al. Salivary DJ-1 could be an indicator of Parkinson's disease progression. Front Aging Neurosci 2014;6:102
